# Correction: Enhanced expression of pro-inflammatory mediators and liver X-receptor-regulated lipogenic genes in non-alcoholic fatty liver disease and hepatitis C

**DOI:** 10.1042/CS-2010-0387_COR

**Published:** 2024-06-19

**Keywords:** chronic hepatitis C virus (HCV) infection, inflammation, lipogenesis, liver X receptor (LXR), non-alcoholic fatty liver disease (NAFLD)

The authors of the original article “Enhanced expression of pro-inflammatory mediators and liver X-receptor-regulated lipogenic genes in non-alcoholic fatty liver disease and hepatitis C” (DOI: 10.1042/CS20100387) would like to correct their paper. The authors state that as a result of incorrect figure assembly, there is an unintentional duplication of the β-actin panel corresponding to HCV and HCV+NAS groups in Figure 2, Figure 4, and Figure 7 of this paper with a β-actin panel of another paper by San-Miguel et al, 2020 (DOI: 10.3945/jn.110.121525), which shares an author.

**Figure 2:**
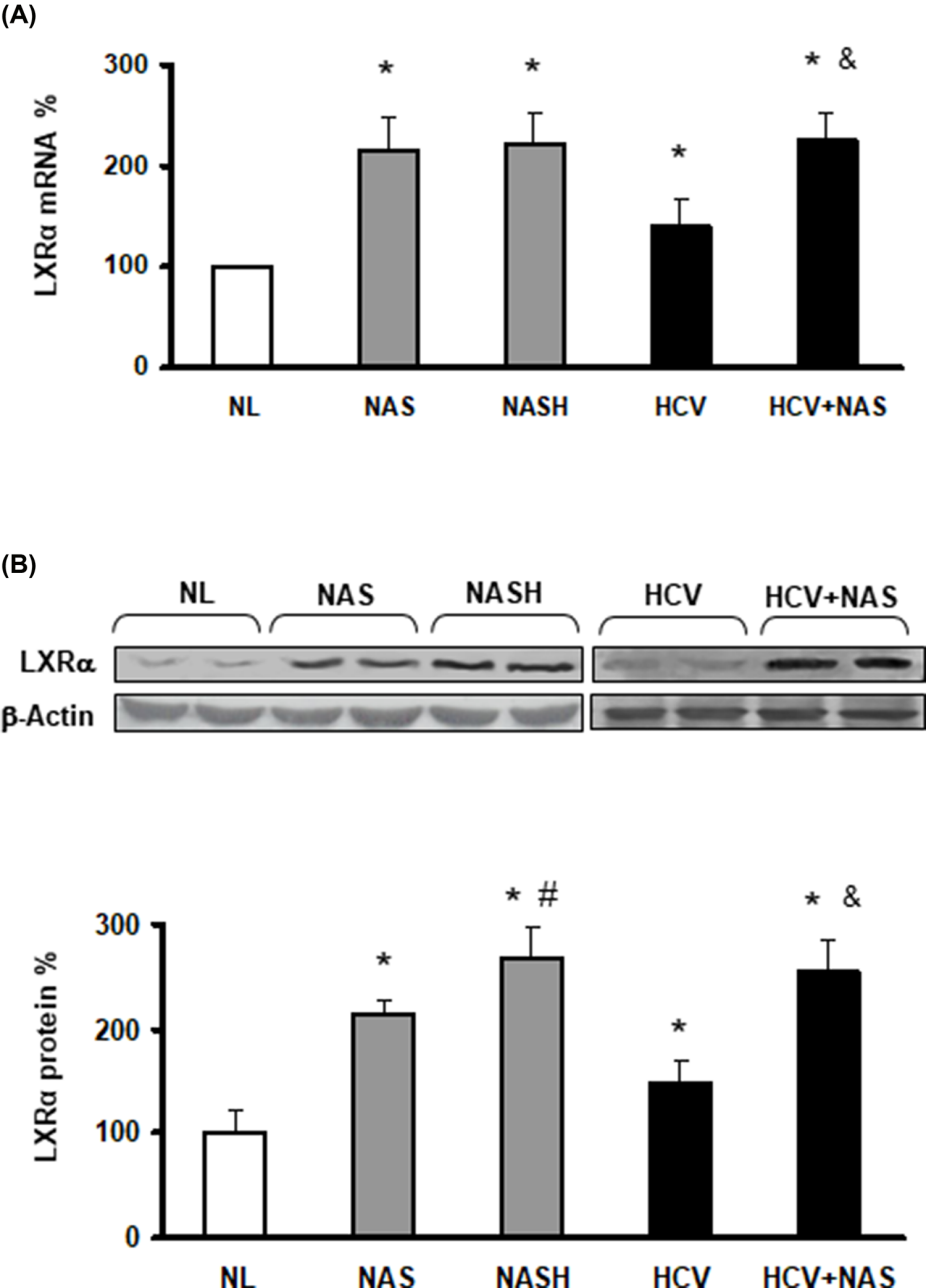
Hepatic overexpression of LXRα in NAFLD and HCV patients

**Figure 4:**
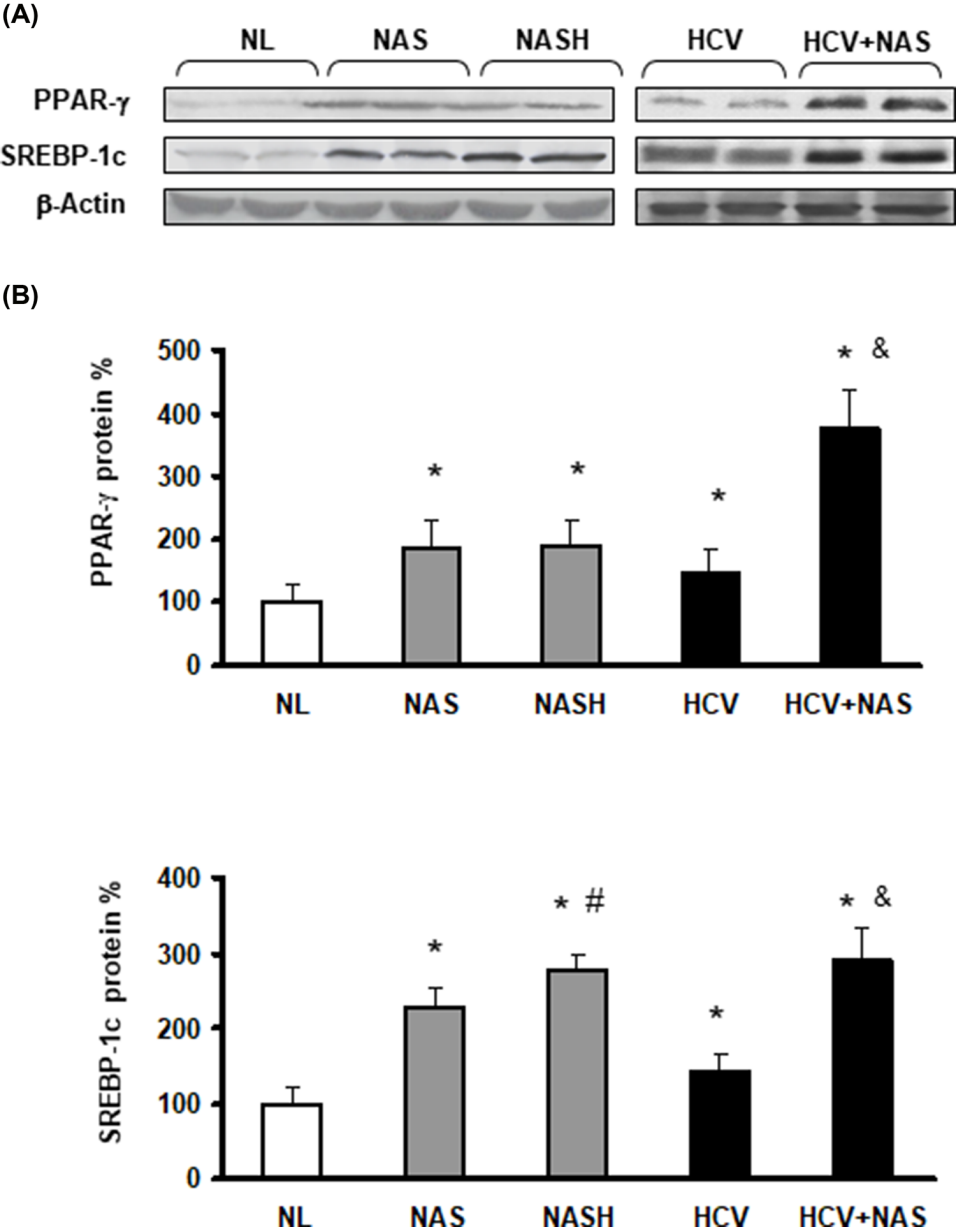
Hepatic content of PPAR-γ and SREBP-1c proteins is also increased in NAFLD and HCV patients

**Figure 7:**
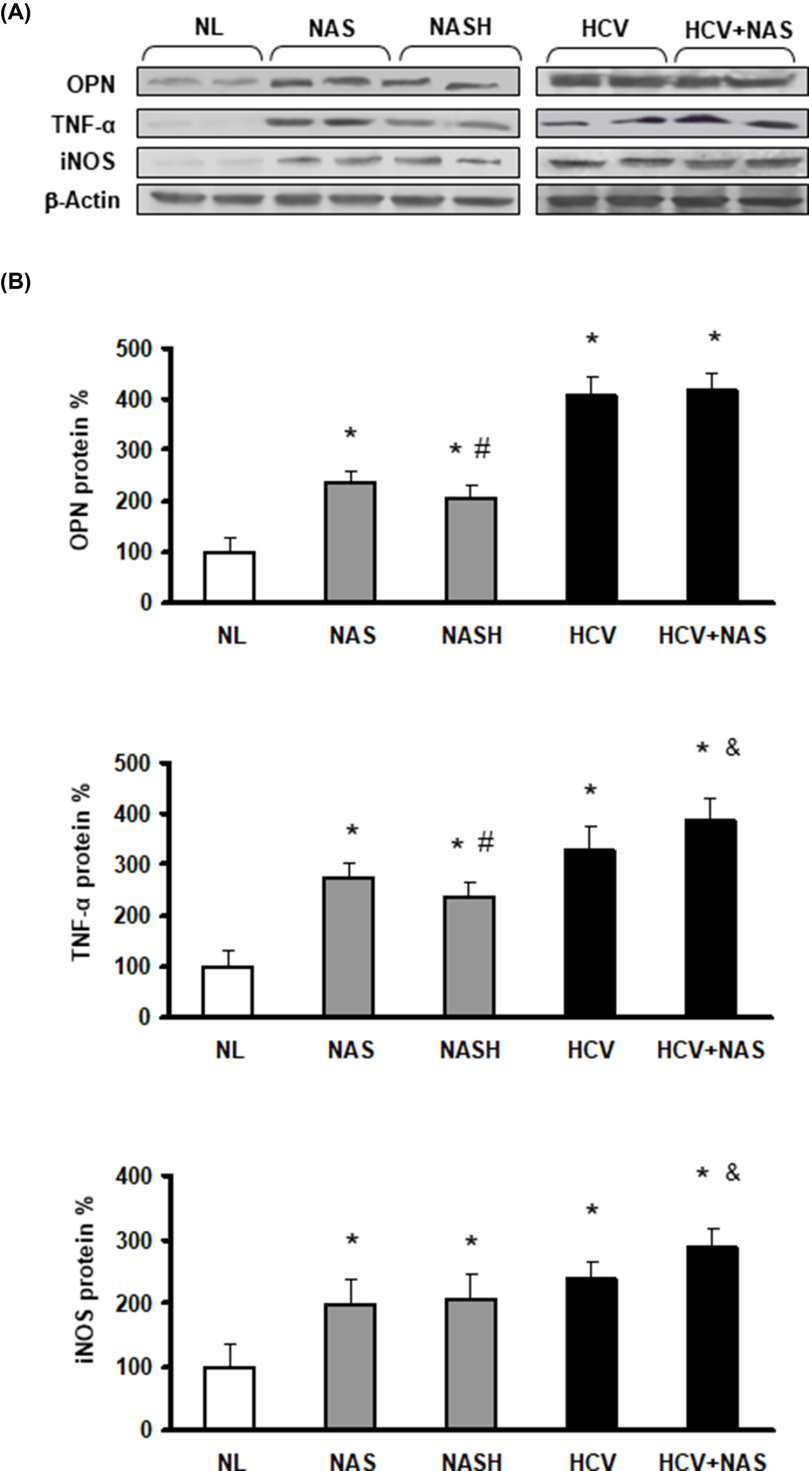
Increased hepatic content of OPN, TNF-α and iNOS proteins in NAFLD and HCV patients

The requested correction has been assessed and agreed by the Editorial Board and Editor-in-Chief. The authors state that no changes to the figure legends are needed, and the correction does not alter the results or conclusions of their paper. The authors would like to apologize for any inconvenience caused. The corrected version of Figure 2, Figure 4, and Figure 7 are presented here.

